# Oncogenic Sox2 regulates and cooperates with VRK1 in cell cycle progression and differentiation

**DOI:** 10.1038/srep28532

**Published:** 2016-06-23

**Authors:** David S. Moura, Isabel F. Fernández, Gema Marín-Royo, Inmaculada López-Sánchez, Elena Martín-Doncel, Francisco M. Vega, Pedro A. Lazo

**Affiliations:** 1 Experimental Therapeutics and Translational Oncology Program, Instituto de Biología Molecular y Celular del Cáncer, Consejo Superior de Investigaciones Científicas (CSIC)-Universidad de Salamanca, Campus Miguel de Unamuno, 37007 Salamanca, Spain; 2 Instituto de Investigación Biomédica de Salamanca-IBSAL, Hospital Universitario de Salamanca, 37007 Salamanca, Spain; 3 Instituto de Biomedicina de Sevilla (IBIS), CSIC-Universidad de Sevilla-Hospital Universitario Virgen del Rocío, 41013 Sevilla, Spain; 4 Departamento de Fisiología Médica y Biofísica, Facultad de Medicina, Universidad de Sevilla, 41013 Sevilla, Spain

## Abstract

Sox2 is a pluripotency transcription factor that as an oncogene can also regulate cell proliferation. Therefore, genes implicated in several different aspects of cell proliferation, such as the VRK1 chromatin-kinase, are candidates to be targets of Sox2. Sox 2 and VRK1 colocalize in nuclei of proliferating cells forming a stable complex. Sox2 knockdown abrogates *VRK1* gene expression. Depletion of either Sox2 or VRK1 caused a reduction of cell proliferation. Sox2 up-regulates *VRK1* expression and both proteins cooperate in the activation of *CCND1*. The accumulation of VRK1 protein downregulates *SOX2* expression and both proteins are lost in terminally differentiated cells. Induction of neural differentiation with retinoic acid resulted in downregulation of Sox2 and VRK1 that inversely correlated with the expression of differentiation markers such as N-cadherin, Pax6, mH2A1.2 and mH2A2. Differentiation-associated macro histones mH2A1.2and mH2A2 inhibit *CCND1* and *VRK1* expression and also block the activation of the *VRK1* promoter by Sox2. VRK1 is a downstream target of Sox2 and both form an autoregulatory loop in epithelial cell differentiation.

Sox2 is a transcription factor that has a major role in cellular reprogramming in cooperation with other factors such as Oct4, c-myc and Klf4^1^. In addition to this role in reprogramming, Sox2 also participates in self-renewal of stem cells as well as in proliferation and specific cell differentiation, which leads to the formation of tissues after several rounds of cell division. Therefore, it is likely that Sox2 has an important role in the regulation of cell proliferation, and in this context its expression also might function as an oncogene.

Sox2 plays several roles in stem, proliferating and differentiating cells^2^. Sox2 has been shown to participate in the regulation of cell proliferation, where it works either as an activator or an inhibitor^3^ and Sox2 is required for self-renewal of stem cells, normal or tumoral^4^, which implies cell division. Furthermore, Sox2 is also implicated in tumor biology. The human *SOX2* gene, located in 3q26.3-q27, is frequently amplified and overexpressed in several types of carcinomas^5^,^6^, which is consistent with its oncogenic role. Expression of Sox2 is an initiating event in the development of squamous cell carcinomas^7^, where it regulates tumor stem cells^8^ and also functions as an oncogene^5^,^9^,^10^. Furthermore, knockdown of Sox2 reduces proliferation of tumor cells from glioblastomas^11^, osteosarcomas^12^, breast cancer^8^ and small-cell lung cancer^9^, which supports an implication of Sox2 in the proliferation phenotype. These observations mean that Sox2 has to establish a functional regulatory link with other proteins implicated in the control of cell proliferation.

VRK1 is a Ser-Thr chromatin kinase that forms complexes with histones^13^,^14^,^15^,^16^,^17^ and transcription factors^18^,^19^,^20^,^21^. Besides, VRK1 is required for several processes in cell division^22^. VRK1 participates in G0 exit and entry in G1^17^,^23^ and its depletion results in a cell cycle arrest^24^. VRK1 is also necessary for chromatin compaction in G2/M^15^, regulation of nuclear envelope assembly and disassembly^25^,^26^, as well as Golgi fragmentation in mitosis^27^ and regulation of Cajal Bodies assembly in cell cycle^28^. In normal epithelia, VRK1 is expressed in a cell subpopulation of the epithelial basal layer^17^, at a frequency consistent with the initiation of cell division by an epithelial stem cell that leads to formation of the proliferation compartment. Expression of VRK1 in squamous epithelia is consistent with this role, where it is present in dividing cells, colocalizing with Ki67^29^ and p63^17^, but not in differentiated cells^17^,^29^. Depletion of VRK1 also causes a reduction in cell proliferation^17^,^30^. This suggested a potential role for VRK1 in the initial asymmetric division of stem cells in which two types of cells are generated. One stem cell, and another cell destined for further expansion and differentiation through mechanisms which also require the activity of VRK1^17^.

Cell proliferation and cell differentiation are processes sequentially organized in tissue formation, and they have different roles. One is associated to expansion of the cellular population, while the other is implicated in the acquisition of the final cell phenotype required to form a tissue, and is an inhibitor of cell proliferation^17^,^29^,^30^. After a cell decides to divide and differentiate there are a series of biological steps that control the switch from division to differentiation. In this context genes controlling cell division are very likely targets of its transcriptional activity, among which VRK1^22^,^31^ is a potential candidate to be regulated by Sox2 since both proteins are associated with the proliferation phenotype. In this report we have studied and characterized the functional interaction between the transcription factor Sox2 and the VRK1 chromatin kinase in the context of cellular proliferation and differentiation.

## Results

### VRK1 and Sox2 colocalize in normal epithelium and in carcinoma cell lines

**Figure 1:**
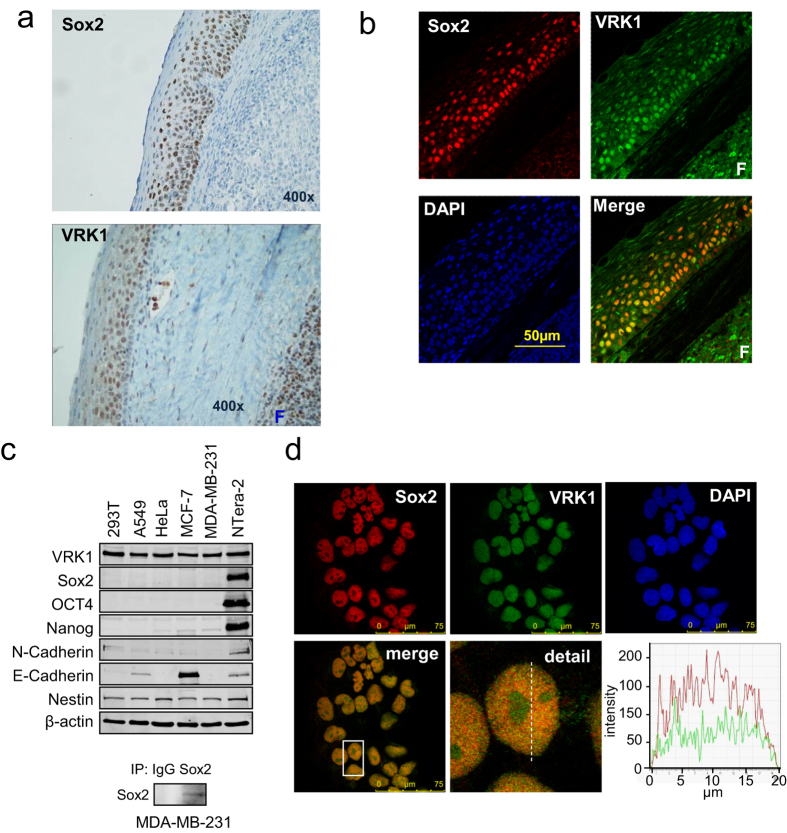
Sox2 and VRK1 expression in normal epithelium and tumor cell lines. (**a**) Expression of Sox2 and VRK1 in stratified epithelium from a normal human tonsil. VRK1 was detected with rabbit polyclonal VC1 antibody, and Sox2 was detected with murine mAb, by immunohistochemistry. (**b**) Sox2 and VRK1 in tonsil epithelium were detected by confocal immunofluorescence. (**c**) Expression of Sox2 and VRK1 protein was detected by immunoblot analysis. In MDA-MB-231 cells Sox 2 was determined after immunoprecipitation of endogenous Sox2 (bottom) with a monoclonal antibody and detected with a polyclonal antibody. (**d**) Colocalization of Sox2 and VRK1 in nuclei of NT2 cells by confocal microscopy. To the right is shown the profile of the signal in the plane. Similar co-localization of Sox2 and VRK1 was detected in human MCF7 and MDA-MB-231cell lines (Supplementary Fig. S1a). F: lymphoid follicle.

The implication of both Sox2 and VRK1 in cell proliferation led us to determine whether there is a relationship between these two proteins. Initially it was determined the pattern of Sox2 and VRK1 protein expression in a stratified squamous epithelium from a normal human tonsil. VRK1 and Sox2 presented a similar localization, manifested by their staining within the amplification compartment, where proliferating cells are located. The expression of both proteins decreased as epithelial cells were terminally differentiated (Fig. 1a). The colocalization of Sox2 and VRK1 in the same nuclei of normal epithelial cells was confirmed by their overlapping staining detected by confocal immunofluorescence (Fig. 1b).

To study the potential relationship between Sox2 and VRK1, we also determined their presence in several cell lines: HEK293T cells, MCF7 and MDA-MB-231 breast cancer cell lines, and N-tera2 (NT2)^32^ cells, a teratocarcinoma cell line that can differentiate into neurons. NT2 cells have high levels of both Sox2 and VRK1 proteins (Fig. 1c, top). Low endogenous levels of Sox2 became detectable in MDA-MB231 cells by immunoblots only after immunoprecipitation (Fig. 1c, bottom). Although expression levels of Sox2 varied among cell lines, nuclear colocalization of Sox2 and VRK1 was confirmed by immunofluorescence in NT2 cells (Fig. 1d), MDA-MB-231 and MCF7 cells (Supplementary Fig. S1a). The nuclear fluorescence of Sox 2 is three fold higher in NT2 cells (Fig. S1b).

### Sox2 forms a stable protein complex with VRK1

**Figure 2:**
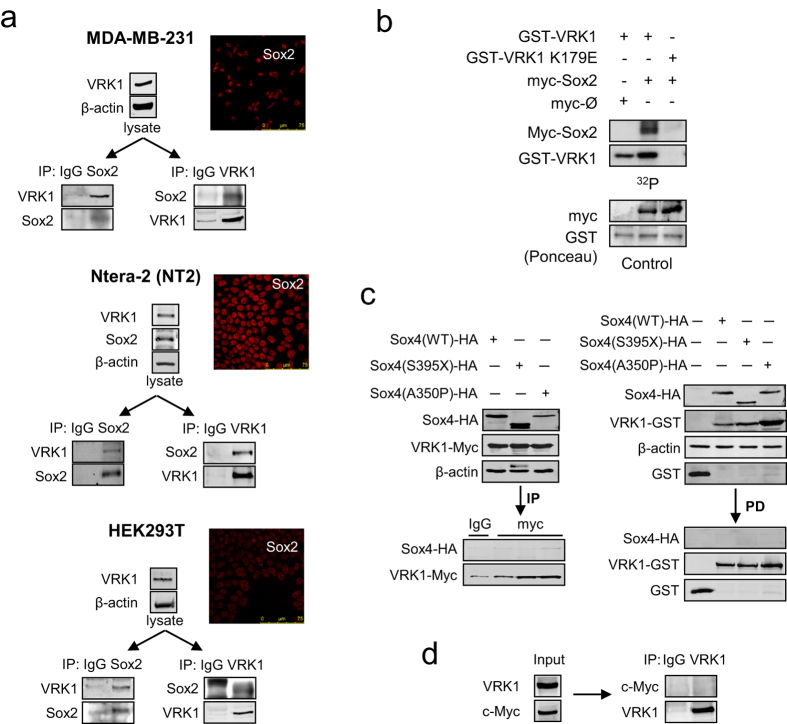
Sox2-VRK1 protein interaction. (**a**) Human endogenous Sox2 interacts with human endogenous VRK1. The VRK1-Sox2 intracellular complex was detected in MDA-MB-231(top), NT2 (center) and HEK293T (bottom) cells. Cell lysates were immunoprecipitated with a mAb anti-Sox2 mAb (E-4) or anti-VRK1 (1B5)^64^. Human endogenous VRK1 was detected in immunoblots with anti-VRK1 (VC1) antibody^64^ and Sox2 was detected with Y-17 antibody. Control immunoprecipitations were done with a nonspecific antibody. The Sox2-VRK1 interaction was confirmed in transfection experiments with tagged HA-VRK1 and Flag-Sox2 (Supplementary Fig. S1b) and in pulldown assays using GST-VRK1 (Supplementary Fig. S1c). (**b**) *In vitro* kinase assay using VRK1 or kinase-dead VRK1 (K179E) with immunoprecipitated myc-Sox2 as substrate. (**c**) Lack of interaction between VRK1 and Sox4. Tagged Sox4 wt and mutants (S395X or A350P) were co-transfected with either VRK1-myc (left) or GST-VRK1 (right) for either immunoprecipitation (IP) or pull down assays (PD) respectively. (**d**) Lack of interaction between endogenous VRK1 and c-myc. Endogenous VRK1 was immunoprecipitated with anti-VRK1 (1B5)^64^.

VRK1 is a chromatin kinase that regulates histones^15^,^16^ and also forms stable complexes with several transcription factors such as p53^19^, ATF2^21^, c-Jun^20^ and CREB1^18^, which are also its phosphorylation targets. Sox2 is a SRY-related HMG-box transcription factor that is phosphorylated in several residues^33^. Therefore, we hypothesized that VRK1 could also be forming a stable protein complex with the Sox2 transcription factor. To detect this possible interaction we performed reciprocal immunoprecipitations of endogenous VRK1 and Sox2 proteins from MDA-MB-231, NT2 and HEK293T cells. In the three cell lines Sox2 was detected in the VRK1 immunoprecipitate, and reciprocally VRK1 was also detected in the Sox2 immunoprecipitate (Fig. 2a). This stable VRK1-Sox2 protein interaction was further confirmed by either immunoprecipitation of transfected tagged-proteins (Supplementary Fig. S2a), and the interaction was also dependent on the amount of transfected protein (Fig. S2b). This interaction Sox2-VRK1 interaction was further confirmed by performing GST-VRK1 pulldown (Supplementary Fig. S2c), or immunoprecipitation experiments (Fig. S2d) and using deletion fragments of the VRK1 protein. Because VRK1 is a Ser-Thr kinase, the potential phosphorylation of Sox2 by VRK1 was demonstrated using kinase active and inactive VRK1. VRK1, but not kinase-dead VRK1(K179E), phosphorylated Sox2 (Fig. 2b). Therefore, VRK1 is one of the kinases phosphorylating Sox2. Sox proteins share an HMG motif but have different structure and functions^34^. Therefore, we also tested if VRK1 can interact with Sox4, which has different roles. An interaction between VRK1 and Sox4 was not detected in immunoprecipitation or GST-pulldown assays (Fig. 2c). In addition, VRK1 also did not interact with endogenous c-myc protein (Fig. 2d), whose expression is regulated by VRK1^17^.

### Depletion of Sox2 results in loss of VRK1 and cell proliferation

**Figure 3:**
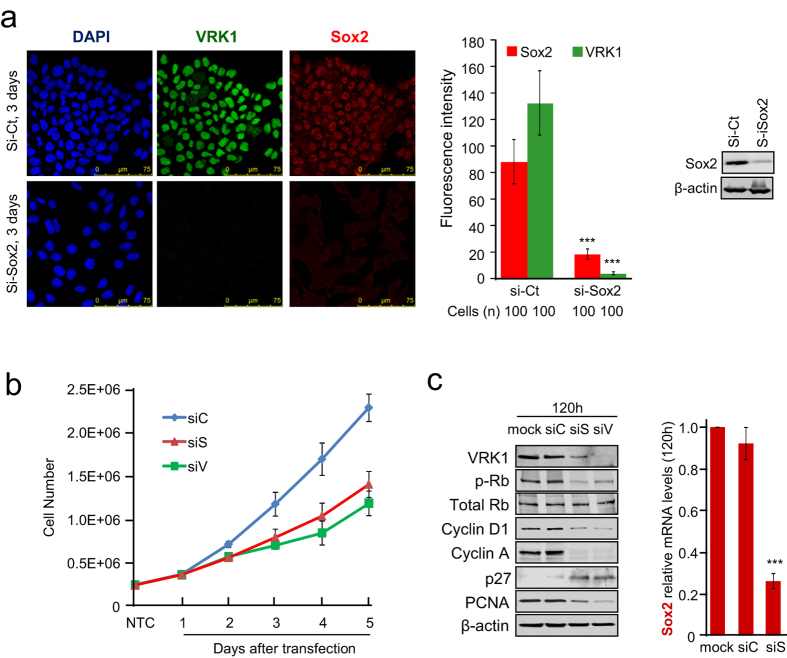
Sox2 knockdown results in loss of VRK1 and of cell proliferation. (**a**) Depletion of Sox2 in NT2 cells. Sox2 was knocked down and the level of Sox2 protein determined by immunofluorescence and western blot. One hundred cells were used for immunofluorescence quantification with the LAS Lite program. Sox2 was detected with a mouse monoclonal anti- Sox2 (E-4, 1:100). VRK1 was detected using a rabbit polyclonal anti-VRK1 antibody (1:200). (Student’s Test: * < 0.05; ** < 0.005; *** < 0.0005). siC: si-control. The western blot with the knockdown of Sox2 is shown to the right. (**b**) Effect of silencing Sox2 or VRK1 on the rate of cell proliferation in MDA-MB-231 cells. An equal number of cells (200 000) were seeded and counted every twenty-four hours (left). The results show the mean of three experiments, in which each point was determined in triplicate. (**c**) Expression of proteins associated to cell cycle progression. Cells were also used to determine the expression of different proteins associated with proliferation (center). Because of the low number of cells the level of Sox2 was determined by qRT-PCR (right). siC: si-control, siS: si-Sox2, siV: si-VRK1-02.

The co-localization of Sox2 and VRK1 in the proliferation compartment, as well as their physical interaction suggested that both might contribute to control of cell proliferation. Fist we determined the effect of Sox2 knockdown on the levels of Sox2 and VRK1 in NT2 cells. Depletion of Sox2 resulted in the loss of Sox2 and VRK1, suggesting that VRK1 can be a downstream target of Sox 2 (Fig. 3a). Sox2 depletion also caused a reduction in cell number by immunofluorescence. Next, because both proteins have been associated to proliferation^17^,^35^, we tested if depletion of either Sox2 or VRK1 in MDA-MB-231 cells can have an effect on cell proliferation. Depletion of Sox2, or VRK1, caused a similar reduction in the rate of cell duplication (Fig. 3b). Depletion of either of these proteins was also manifested by the loss of phospho-Rb, cyclins D1 and A, PCNA, all them associated to active cell proliferation (Fig. 3c). Depletion of Sox2, or VRK1, also resulted in accumulation of p27, an inhibitor of cell cycle progression (Fig. 3c).

### Sox2 activates *VRK1* gene expression

**Figure 4:**
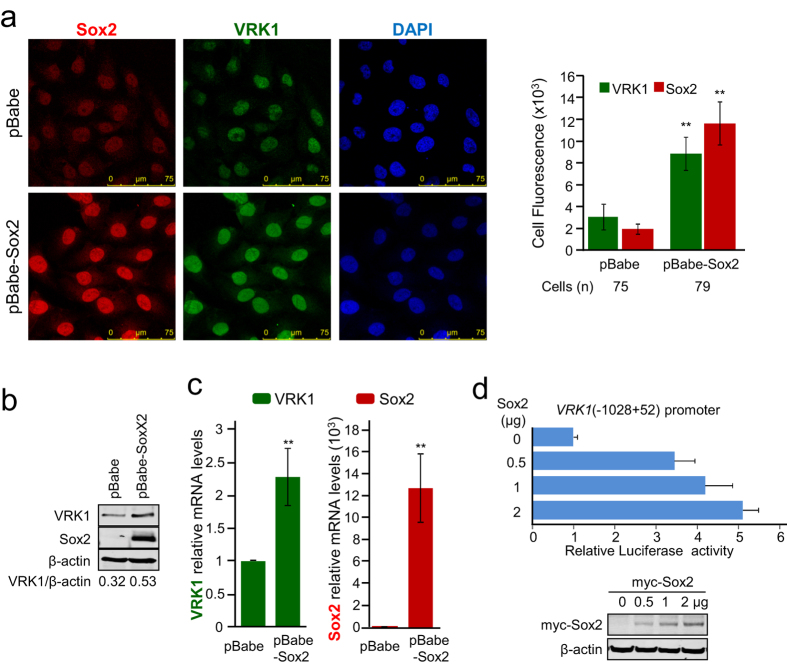
Sox2 activates endogenous *VRK1* gene expression. (**a**) Effect of Sox2 overexpression in MDA-MB-231 cells infected with the retroviral plasmid pBabe-Sox2. Cells were studied by confocal immunofluorescence and the quantification of the fluorescence is shown in the graph (right). (**b**) Effect of Sox2 overexpression on endogenous VRK1 protein in cell lysates of MDA-MB-231 cells determined by immunoblot. (**c**) Levels of endogenous VRK1 RNA from control or infected MDA-MB-231 cells with pBabe-Sox2. Sox2 and VRK1 RNA levels were determined by qRT-PCR. (**d**) Effect of overexpression of Sox2 on the proximal *VRK1* promoter (−1028 + 52) in a luciferase reporter construct. At the bottom is shown the expression of transfected Sox2. Reporter gene experiments were performed three times, and each individual luciferase assay was determined in triplicate.

The regulation of *VRK1* gene expression by Sox2 was initially studied in MDA-MB-231 cells that were infected with a retroviral construct overexpressing Sox2. High levels of Sox2 induced an increase of endogenous VRK1 protein that was detected by immunofluorescence (Fig. 4a) and by immunoblot analysis (Fig. 4b). In both approaches, the increase of VRK1 levels was similar. This effect on VRK1 might be due to either increased gene expression or protein stability. The effect of Sox2 overexpression caused an increase in expression of VRK1 mRNA levels (Fig. 4c). A similar effect of Sox2 overexpression on *VRK1* expression was observed in MCF-7 cells (Supplementary Fig. S3a–c). These results suggested that Sox2 is regulating the *VRK1* gene.

Because Sox2 is a transcription factor, we next tested whether the *VRK1* (−1028 to +52) proximal gene promoter, cloned in a luciferase reporter plasmid, was regulated by Sox2. The proximal *VRK1* gene promoter was activated by overexpression of Sox2 (Fig. 4d). A similar result was obtained with HEK293T cells in which the *VRK1* (−1028 + 52) gene promoter was also activated in a dose dependent manner by Sox2 (Supplementary Fig. S3d). However, Sox2 has no effect on the activation of VRK1 promoter comprised between residues −264 to +52 (Supplementary Fig. S4). This shorter promoter region shows a higher level of expression, reflecting the lack of some regulatory elements. These results indicated that the effect of Sox2 on *VRK1* promoter is mediated by the region comprised between residues −1028 to −264.

### Sox2 and VRK1 cooperate in the activation of *CCND1* gene expression

**Figure 5:**
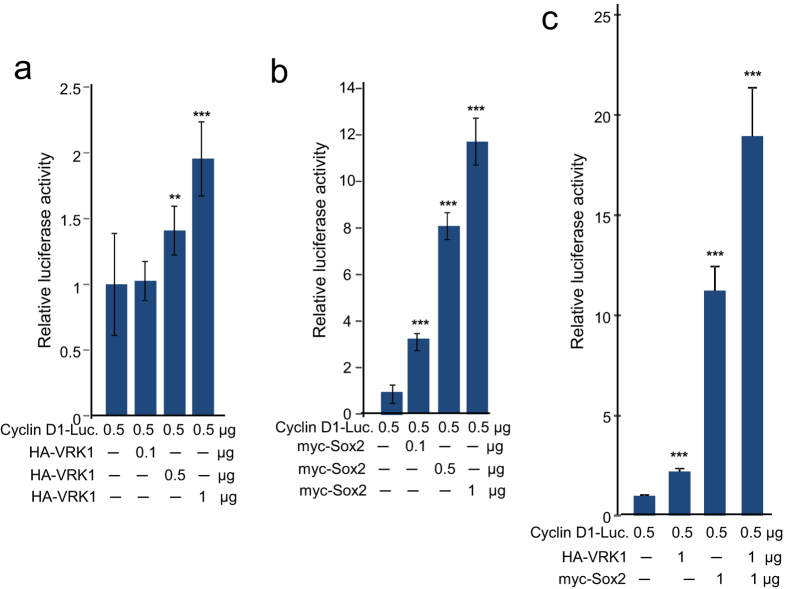
Sox2 and VRK1 cooperate in the activation of the human cyclin D1 (*CCND1*) promoter. Breast carcinoma MDA-MB-231 cells were transfected with 1 μg of pA3-CyclinD1-Luc reporter that contains the proximal promoter (−1745 + 134) and increasing amounts of plasmids expressing Sox2 and VRK1. Forty-eight hours after transfection, cell lysates were prepared and used for determination of luciferase activity. The results are the mean of three independent experiments in triplicate.

Both Sox2^8^ and VRK1^18^ have been implicated in the regulation of *CCND1* gene expression, coding for cyclin D1, which is necessary for cell cycle progression. Sox2 is a transcriptional factor that initiates cell division in the stem cell compartment^7^ and is a known regulator of the G1/S transition in cell cycle, which is mediated by cyclin D1^8^. VRK1 also facilitates cell cycle progression and is able to activate the *CCND1* gene promoter^29^, an effect that is mediated by phosphorylation of CREB1^18^, and its depletion causes a cell cycle arrest in G0/G1^17^. Therefore, we tested whether VRK1 and Sox2 can cooperate in the activation of the proximal *CCND1* gene promoter. Sox2 and VRK1 individually caused an activation of the *CCND1* gene promoter (Fig. 5a,b), which is approximately five fold stronger in the case of Sox2 (Fig. 5b). But the combination of both Sox2 and VRK1 resulted in a higher activation of *CCND1* gene expression (Fig. 5c), which was consistent with a synergistic effect. Therefore, we concluded that Sox2 and VRK1 proteins synergistically cooperate in the activation of *CCND1* gene expression and thus can contribute together to cell cycle progression and proliferation.

### VRK1 levels modulate *SOX2* gene expression

**Figure 6:**
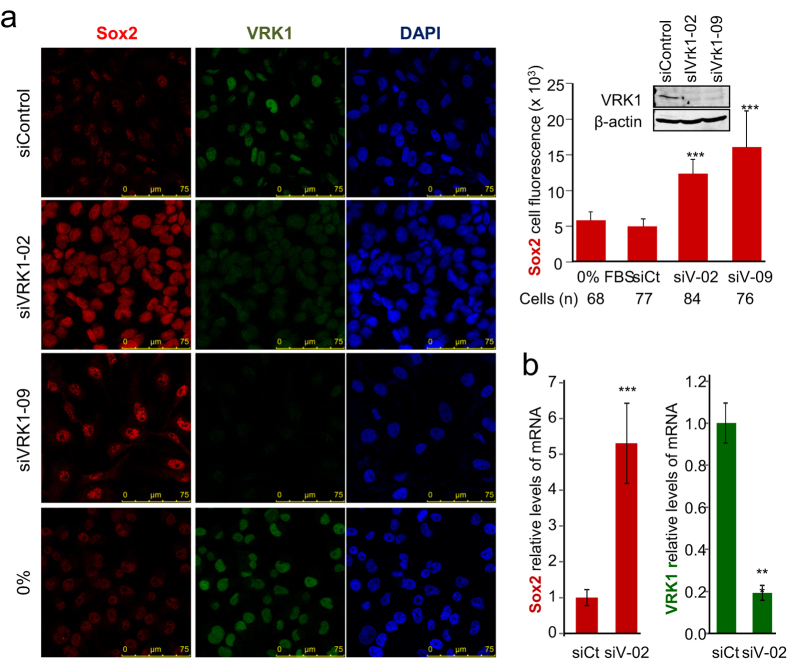
VRK1 depletion upregulates *SOX2* gene expression. (**a**) In MDA-MB-231 cells, human endogenous Sox2 levels increase, when VRK1 levels decrease. 72 hours after siControl, si-VRK1-02 and si-VRK1-09 treatment, was performed the fixation and permeabilization of MDA-MB-231 cells. By immunofluorescence, human endogenous Sox2 was detected with a mouse monoclonal anti-Sox2 (E-4, 1:100). Human endogenous VRK1 was detected using a rabbit polyclonal anti-VRK1 antibody (1:200). DAPI (1:1000). In western blots, human endogenous VRK1 was detected with anti-VRK1 (1B5, 1:1000). Human endogenous Sox2 was detected using a goat polyclonal anti-Sox2 (Y-17, 1:500) and human β-actin was detected using a mAb anti-β-actin (1:5000). (**b**) VRK1 depletion caused a reduction of Sox2 mRNA detected by qRT-PCR. The experiment was determined three times in triplicate determinations. siCt: siControl; siV-02: siVRK1-02; siV-09: siVRK1-09.

The activity of VRK1 is controlled by an autoregulatory loop with some of its targets as has been described in the context of p53^36^,^37^. Therefore, we explored whether there is a cross regulation between VRK1 and Sox2 expression. Initially, the effect of VRK1 depletion on the level of Sox2 was determined by immunofluorescence. Depletion of VRK1 with two different siRNA resulted in an increased of the intracellular level of Sox2 protein in MDA-MB-231 cells (Fig. 6a). In cells that were arrested by serum deprivation for forty eight hours there was no effect on the level of endogenous Sox2 protein (Fig. 6a). This indicated that the effect on VRK1 is not an indirect consequence of his role in arresting cell cycle^17^. The effect is dependent on the kinase activity of VRK1 since VRK1 is kinase-inactive in the absence of serum^38^.

To determine whether the effect of VRK1 depletion on Sox2 protein levels was due to altering *SOX2* gene expression we determined its mRNA levels by qRT-PCR. VRK1 depletion resulted in a significant increase in Sox2 mRNA (Fig. 6b). Similar results were obtained in MCF7 (Supplementary Fig. S5) and NT2 cells (Supplementary Fig. S6).

**Figure 7:**
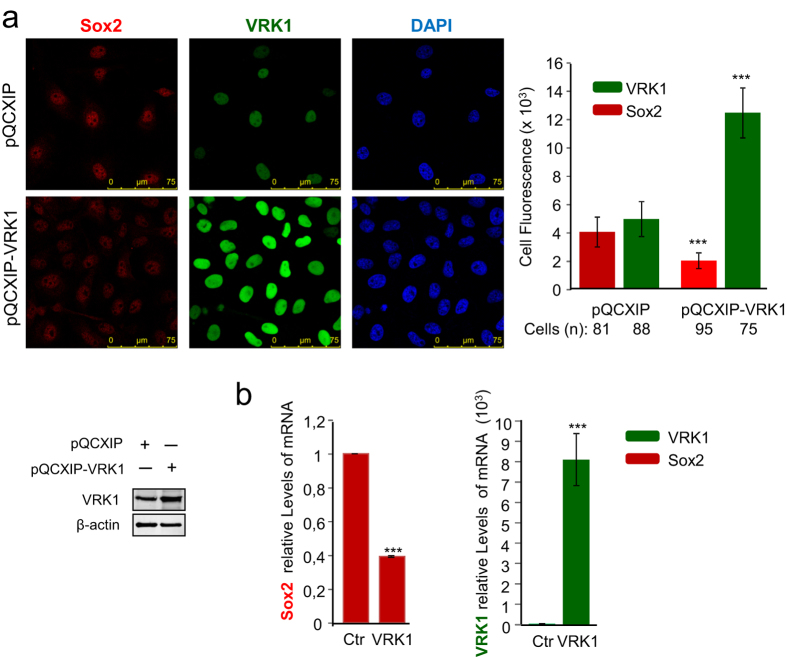
VRK1 overexpression downregulates SOX2 gene expression. (**a**) MDA-MB-231 cells were transduced with pQCXIP (empty vector) or pQCXIP-VRK1 and selected with puromycin. The presence of Sox2 and VRK1 proteins in nuclei was determined by confocal microscopy (left) and the cell florescence was quantified (right). Sox2 was detected with a mouse monoclonal anti-Sox2 (E-4, 1:100). VRK1 was detected using a rabbit polyclonal anti-VRK1 antibody (1:200). VRK1 was detected, using a mouse monoclonal anti-VRK1 (1B5, 1:1000). β-actin was detected using a mouse monoclonal anti-β-actin antibody (1:5000). (**b**) The levels of Sox2 and VRK1 RNA were determined by qRT-PCR in control (pQCXIP) and VRK1 transduced cells (pQCXIP-VRK1) cells. The effect occurs also at mRNA level.

To further confirm the effect of VRK1 on the regulation of *SOX2* gene expression, the opposite experiment was performed. VRK1 was overexpressed using a retroviral construct resulting in downregulation of Sox2 and a loss of Sox2 nuclear fluorescence (Fig. 7a). This effect was the consequence of a reduction in the level of Sox2 mRNA (Fig. 7b). This effect was also confirmed using a stable HeLa cell line overexpressing VRK1 (Supplementary Fig. S7). Therefore, the combined results from knockdown and overexpression experiments indicated that there is a cross regulation between Sox2 and VRK1 and that VRK1 contributes to the downregulation of *SOX2* gene expression.

### Expression of Sox2 and VRK1 in NT2 cell differentiation induced by retinoic acid

**Figure 8:**
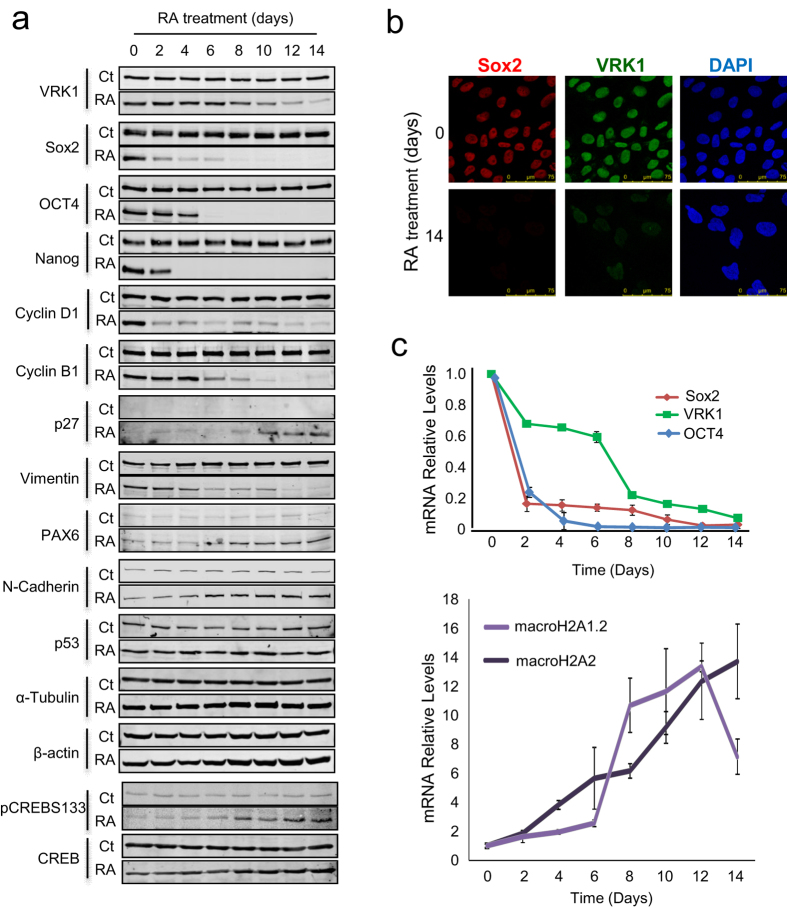
Sox2 and VRK1 levels are downregulated during NT2 cell differentiation induced by retinoic acid. (**a**) Time course of the expression of different proteins in NT2 cells induced to differentiate with retinoic acid (RA). At each time point cells were lysed and the extracts were loaded in 10% acrylamide gel and analyzed by immunoblots. The quantification of the proteins in the immunoblots is shown in Supplementary Fig. S9. (**b**) The expression of Sox2 and VRK1 proteins in NT2 cells was detected by immunofluorescence before and after induction of differentiation with retinoic acid (RA). Sox2 was detected using a mouse mAb anti-Sox2 (E-4, 1:100). VRK1 was detected using a rabbit polyclonal anti-VRK1 (1:200) antibody. (**c**) Effect of RA-induced differentiation of NT2 cells on the gene expression levels Sox2, Oct4, VRK1 and macro H2A2. Total RNA was extracted at the indicated times and the RT-PCR was performed using specific primers indicated in the methods section.

Sox2 plays and important role in the early decision of stem cells^7^, and VRK1 has a role in initiation of cell proliferation^17^,^22^,^30^. Since initiation of lineage differentiation requires an asymmetric cell division we decided to study the fate of Sox2 and VRK1 along the NT2 cell differentiation process induced with retinoic acid. Induction of cell differentiation resulted in a significant loss of stem cell markers including Sox2, Oct4, and Nanog: and also of VRK1, cyclins D1 and B1 proteins, which represent markers of proliferation (Fig. 8a). In addition differentiation markers such Pax6, N-cadherin, p27 and phospho-CREB are increased during RA-induced differentiation as expected. Also markers that are lost during RA-induced differentiation such as vimentin were determined (Fig. 8a). All of the markers studied presented the expected variation as differentiation progressed in time (Fig. 8a). The reduction in levels of Sox2, VRK1 (Fig. 8b), Oct4 and Nanog protein levels (Supplementary Fig. S8) were also confirmed by the loss of their nuclear fluorescence. The differentiation state of NT2 cells was also confirmed by the morphological reorganization of α-tubulin induced by RA (Supplementary Fig. S8). To determine whether these changes in protein levels were due to modifications in gene expression we also determined, by qRT-PCR, the expression of *SOX2*, *OCT4*, *VRK1, H2AFY,* and *H2AFY2*. The two latter genes code for macrohistones mH2A1.2 and mH2A2 that inhibit cell proliferation and are associated with terminal differentiation^39^,^40^. Retinoic acid (RA) induced a downregulation of SOX2, OCT4 and VRK1 mRNA; in addition RA also caused an upregulation of macroH2A1.2 and macroH2A2 mRNA to very high levels in the last days of the differentiation process (Fig. 8c). The differentiation phenotype of NT2 cells was confirmed by detecting the corresponding increase in N-cadherin, βII-tubulin, Pax6 and CREB phosphorylated in Ser133 (Fig. 8a). Thus there is an inverse temporal correlation between the loss of markers regulating cell stemness and proliferation and the increase of those markers associated with cell differentiation.

### Inhibition of *VRK1* and *CCND1* gene expression by macroH2A histones

**Figure 9:**
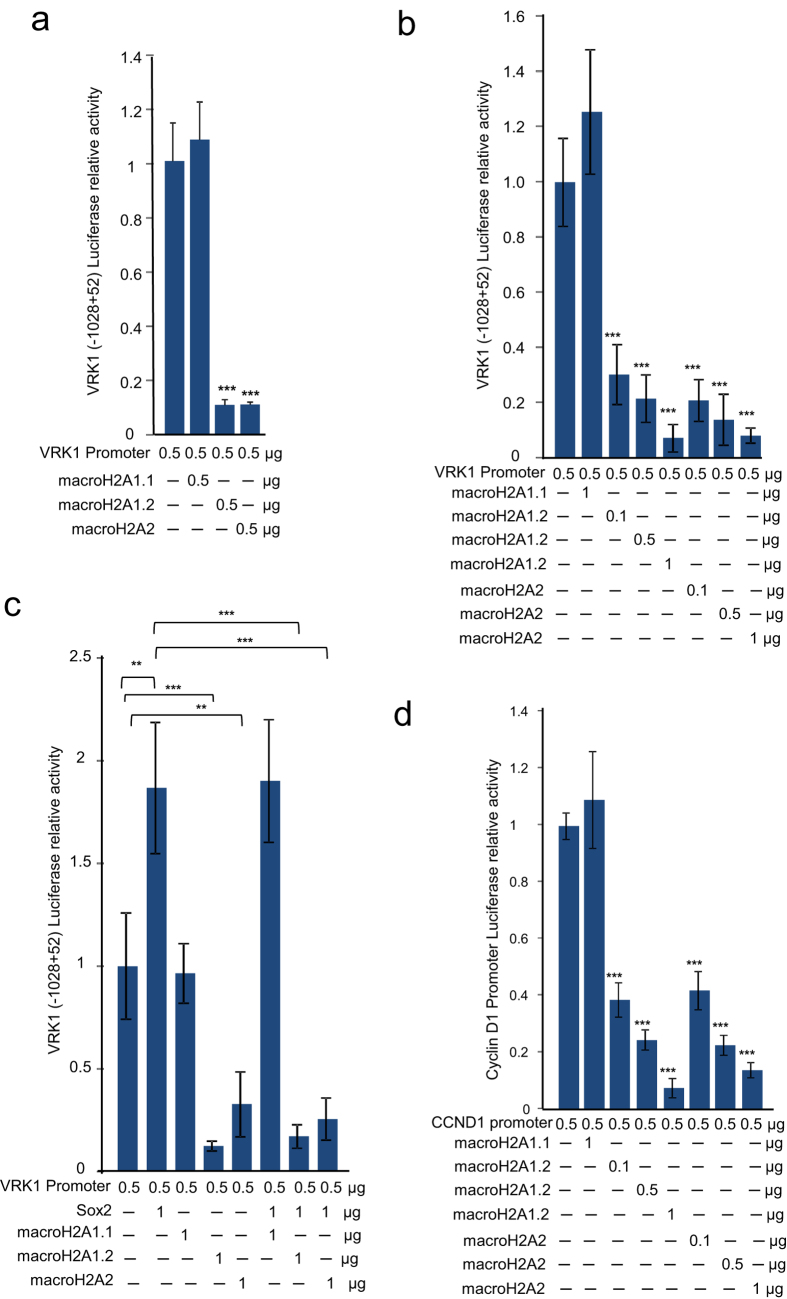
Effect of the three macro-histones H2A on transcription of the *VRK1* and *CCND1* gene promoters. (**a**) Effect of the three macro histones on the proximal promoter of *VRK1* (−1028 + 52) detected in luciferase assays. (**b**) Dose dependent effect of macro-histones H2A on the *VRK1* (−1028 + 52) detected in luciferase assays. (**c**) Effect of macro-histones H2A on the activation of the VRK1 (−1028 + 52) promoter induced by Sox2 and detected in luciferase assays. (**d**) Effect of the three macro histones on the proximal promoter of *CCND1* (−1720 + 134) detected in luciferase assays. All experiments were performed three times and in each experiment determinations were made in triplicate. The mean and standard deviation are shown.

The downregulation of stem genes concomitant with differentiation are mediated by an exchange of histones in which histone H2A is replaced by macro-histones that have a larger C-terminal region^41^,^42^. Macro-histones H2A (mH2A) are histone variants that are associated with differentiated cells and inhibit gene transcription^43^,^44^, as shown for mH2A1.2 and mH2A2 (Fig. 8c). Also macro histone H2A1.2 is known to inhibit the activity of VRK1 by their direct physical interaction^14^. Therefore, it was tested if macro-histones were able to regulate the *VRK1* and *CCND1* gene promoters. The differentiation associated mH2A1.2 and mH2A2 inhibited *VRK1* gene promoter in a dose dependent manner (Fig. 9a,b). However, the proliferation associated mH2A1.1 did not inhibit the *VRK1* promoter. Next, we determined if these macro-histones were also able to inhibit the effect of Sox2 on the *VRK1* gene promoter. The differentiation-induced mH2A1.2 and mH2A2 proteins were able to block the activating effect of Sox2 on the *VRK1* proximal promoter (Fig. 9c). Finally, we also showed that mH2A1.2 and mH2A2, but not mH2A1.1, also inhibited the *CCND1* promoter in a dose dependent manner (Fig. 9d). Therefore, we concluded that macro-histones associated with differentiation inhibit the proliferative signals acting on the *VRK1* and *CCND1* promoters, as well as prevent the effect of Sox2 on *VRK1* expression.

## Discussion

The expression of Sox2 within the amplification compartment of the epithelium suggested that Sox2 has a role in the control of cell proliferation, particularly since it also colocalizes with VRK1 in these cells, which has also been associated to the p63^17^ and Ki67^29^ proliferation markers. This Sox2 expression in tonsil stratified epithelium is analogous to its expression in the pituitary gland^45^ and esophagus epithelium^2^. The interaction between Sox2 and VRK1 in nuclei is consistent with the known interaction of VRK1 with several transcription factors such as p53^19^, ATF2^21^, CREB^18^ or c-Jun^20^, as well as the p300 acetyltransferase transcriptional coactivator^46^. In addition, the phosphorylation of Sox2 by VRK1 can contribute to the regulation of its transcriptional activity or its integration in nuclear complexes with other nucleoproteins and will require further studies.

As cells initiate their first self-renewal asymmetric division it is necessary the cooperation of Sox2 with other proliferation associated proteins, among which the VRK1 is a potential candidate. At this stage Sox2 connects with VRK1, a chromatin kinase, associated to several aspect of cell proliferation^22^ as well to chromatin remodeling^16^. Thus, the regulation of *VRK1* gene by Sox2 is fully consistent with a role for VRK1 as an initiator of cell proliferation^17^. Sox2 increases *VRK1* gene expression by targeting its proximal promoter, but neither Sox2 nor VRK1 are direct regulators of cell cycle initiation and progression. Therefore they have to regulate key genes or proteins implicated in the regulation of cell cycle, among which *CCND1* is a major candidate^47^. Thus, Sox2 and VRK1, as proliferation regulators, synergistically cooperate in the control of cell cycle specific genes, as is the case of *CCND1* that is regulated by both Sox2^35^ and VRK1^18^. *CCND1* gene expression is in part regulated by the CREB transcription factor^18^. In this context, it is important to note that *CREB* gene expression is regulated by Sox2^48^ and CREB protein is phosphorylated and activated by VRK1^18^. Therefore, it is likely that depending on levels of CREB-Ser133 phosphorylation there are different threshold of responses. In dividing cells low levels of phospho-CREB are required for cell cycle progression. But in cells that are differentiated and in which a different set of genes are required to fulfill their specific functions, a higher level of phosphorylation may be necessary. Thus, it is possible that in differentiated cells, Ser133 phosphorylation is mediated by different kinases, among which are calcium/calmodulin-dependent protein kinase types II and IV^49^. Therefore, CREB phosphorylation can have different roles depending on whether it is participating in cell cycle progression or in differentiation.

Accumulation of VRK1 protein also negatively down regulates Sox2, since cells are already determined, but proliferation is still necessary. The lower rate of VRK1 downregulation in differentiation is a consequence of the high stability of this protein^17^. As cells differentiate the accumulation of VRK1 protein can inhibit Sox2 expression, and also mH2A1.2 can inhibit the kinase activity of VRK1^14^ that facilitate cell differentiation.

Differentiated cells do not divide and this implies the need for a downregulation of proliferative proteins. In this context as VRK1 reaches a high level, it is able to activate *CCND1* by itself and also down-regulate *SOX2* by an unknown mechanism but that is likely to include regulation of the transcriptional complex. This latter effect is consistent with the upregulation of pluripotency transcription factors, Sox2, Oct4 and c-myc, caused by luteolin, an inhibitor of VRK1^50^. Moreover, high VRK1 can stabilize p53^19^,^51^ and contribute to the necessary cell cycle arrest to permit differentiation. Inhibition of proliferation and facilitation of differentiation is mediated by changes in chromatin and this change is associated to incorporation of histone variants. For this remodeling of chromatin in differentiation, the role of macrohistones mH2A1.2 and H2A2 is very important because they inhibit the VRK1 promoter, and also prevent the effect of Sox2 on the *VRK1* and *CCND1* promoters.

The role of VRK1 within the tumor phenotype is complex, since it can function as an oncogene or tumor suppressor depending on the cellular context. VRK1 is strong facilitator of cell cycle progression^15^,^17^ and proliferation^17^,^30^, and many tumors have very high levels of VRK1 protein which is consistent with an oncogenic function of wild-type VRK1. High levels of VRK1^52^,^53^ and Sox2^54^,^55^ are markers of a poorer prognosis in breast cancer, which is a reflection of their contribution to expanding the tumor cell population. But VRK1 is also positive regulator of p53^19^,^24^,^51^,^56^, and contributes to genomic stability by facilitating DNA damage responses^16^,^38^,^57^. In this latter context, the role of VRK1 is similar to that of other tumor suppressor genes or tumor predisposition genes. Thus, the complexity of VRK1 functions might be a reflection of its late appearance in evolution as organisms become more complex, and where VRK1 appears to be implicated in the coordination of diverse signaling processes and functions in vertebrates.

**Figure 10:**
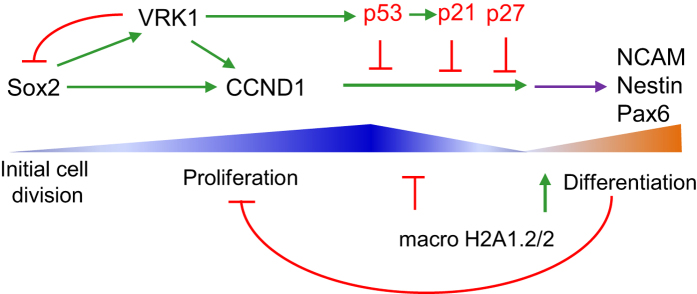
Diagram of the Sox2 and VRK1 roles in the regulation of cell proliferation and differentiation. As cells enter proliferate there is an increase in Sox2 levels that activate VRK1 and CCND1 gene expression. In this activation also participates VRK1. As cells enter the differentiation progress there is an accumulation of cell cycle inhibitors, a reduction of Sox2 and VRK1 as well as an increase in terminally differentiation markers, such as NCAM, nestin and Pax6. In differentiated cells there is also a change in the pattern of macro histones and an accumulation of macro H2A1.1 and macro H2A2. The triangles represent the increase or decrease of different markers; for Sox2, VRK1 and CCND1 in blue, and for NCAM, Nestin and Pax6 in orange. Red lines indicate inhibition, and green lines indicate activation.

In this work we have identified a functional connection between Sox2 and *VRK1*. Initially Sox2 activates VRK1 gene expression and both proteins synergistically activate *CCND1* expression that promotes cell proliferation. In addition VRK1 contributes to *SOX2* downregulation to reduce proliferation and facilitate differentiation to which macrohistones contributes by completely shutting down Sox2 and VRK1 in terminally differentiated cells. But in the process of differentiation both are downregulated. The data shows a coordinated role for these proteins in the transition from cell division to cell differentiation (Fig. 10).

## Material and Methods

### Plasmids

For overexpression of human VRK1, cells were infected with pQCXIP-*VRK1*, retroviral plasmid containing full-length human VRK1, and used as a pool. Plasmid expressing VRK1, pCEFL-HA-VRK1 has been described^27^,^28^,^51^,^58^. The proximal promoter of human *VRK1* (−1028 to +52 and −274 to +52) were cloned in pGL2-basic (Promega, WI). Human *CCND1* gene promoter was studied with pA3-CyclinD1-Luc (wt −1745 + 134)^59^. Plasmids expressing macroH2A1.1 macroH2A1.2 and macroH2A2 were from M. Buschbeck^43^. Sox2: pCMV6-myc-Flag-Sox2 (Origene; Rockville, MD) or pBabe-Sox2. Sox4: pCDNA3.1-Sox4, pCDNA3.1-Sox4(S395X), and pCDNA3.1-Sox4(A350P)^60^.

### Cell lines and Transfections

The following validated cell lines were used: HeLa, HEK293T, MCF7, MDA-MB-231 and NTera2(NT2)^61^ (ATCC-LGC, Teddington, UK). Cell lines were cultured in (DMEM) medium (Sigma-Aldrich; St-Louis, MO) with 10% fetal calf serum. For transient transfections, cells were transfected using polyethylenimine (PEI) reagent (Polysciences, Inc.; Warrington, PA) as reported^27^,^62^,^63^.

### Knockdown of VRK1

VRK1 knockdown was performed using three different siRNA for VRK1 (NM_003384): siVRK1-02 (siV1-02), siVRK1-03 (siV1-03) and siVRK1-09 (siV1-09) (Dharmacon RNA Technologies,). The sequences targeted by these VRK1 siRNA oligonucleotides were siVRK1-01: GAAAGAGAGTCCAGAAGTA; siVRK1-02: CAAGGAACCTGGTGTTGAA; si-VRK1-03: GGAAUGGAAAGUAGGAUUA; and siVRK1-09: AGGUGUACUUGGUAGAUUA. Sox2 was knockdown with the SOX2 Trilencer-27 human siRNA (Origene, Rockville, MD). As negative control the “ON-TARGETplus siCONTROL Non-targeting siRNA” (siCt) (Dharmacon) was used. The efficiency of RNAi transfection was determined with “siGLO RISC-free siRNA” (DHARMACON) labelled with a red fluorochrome. All of them have been previously used. Cells were transfected with the indicated siRNA (20 nM), using Lipofectamine 2000 (Invitrogen; Carlsbad, CA) as previously reported^16^,^17^,^28^,^38^,^57^.

### Antibodies

The antibodies used were: policlonal VRK1 (VC) and monoclonal VRK1 (1B5 or 1F6)^64^. VRK1 (HPA000660, Sigma). Sox2 (D6D9, Cell Signaling; Berverly, MA,; E4, Santa Cruz; Santa Cruz, CA; Y-17, Santa Cruz,). Oct4 (2840, Cell Signaling), Nanog (D73G4, Cell Signaling), N-Cadherin (H-63, Santa Cruz), Vimentin (RV202, Abcam; Cambridge, UK). Flag epitope (Sigma-Aldrich, monoclonal M5; Sigma-Aldrich, polyclonal F7425). HA epitope (Santa Cruz, F-7; Sigma-Aldrich, H6908), myc epitope (06–549, Millipore; Billerica, MA; 05–724, Millipore), GST (B-14, Santa Cruz). Cyclin D1 (M-20, Santa Cruz). Cyclin A (C-19, Santa Cruz). Rb (C-15, Santa Cruz). Phospho-Rb (Ser807/811)(9308, Cell Signaling). PCNA (PC10, Santa Cruz). PAX6 (PRB-278P, Covance; Princeton, NJ), p27 (610241, BD-Transduction Laboratories; Franklin Lakes, NJ). CREB (9104, Cell Signaling). Phospho-CREB (Ser133) (9191, Cell Signaling). C-myc (N-262, Santa Cruz). β-actin (AC-15, Sigma-Aldrich). Secondary antibodies goat α-Mouse IgG, DyLightTM 680 and/or goat α-Rabbit Ig-G, DyLightTM 800 (Thermo Fischer Scientific) were used for detection in a Li-Cor Odyssey system (Thermo Fisher Scientific; Waltham, MA).

### Immunofluorescence and confocal microscopy

Cells were plated on 35 mm dishes with several coverslips, one for each antibody to be used to avoid well variation, and transfected 24 hours later with expression plasmids or siRNA. Two or three days after, respectively, the slides were collected and the cells were fixed with 3% paraformaldehyde for 30 min at room temperature. Then the cells were treated with a solution of glycine 200 mM for 15 min, and then permeabilized with PBS 0,2% Triton X100 for 30 min. The cells were blocked with 1% BSA in PBS for 1 hour. Proteins of interest were detected by immunostaining with specific antibodies that were incubated overnight or for 1 hour depending on commercial instructions. The secondary antibodies were incubated together (goat anti-mouse Cy3 or Cy2 and goat anti-rabbit Cy3 or Cy2 from Jackson ImmunoResearch) for 1 hour at room temperature. Finally cells were stained with DAPI (4′,6′-diamidino-2-phenylindole) (Sigma) 1:1000 in PBS for 10 min at room temperature, the cells were washed with PBS and slides were mounted with Mowiol (Calbiochem-Merck, Darmstadt Germany), and analysed with a LEICA SP5 DMI-6000B confocal microscope. The lasers used were: Argon (488 nm), DPSS (561 nm) and UV Diode (405 nm). Images were captured with a 63.0x lens zoomed in 1.5–3× with a 1024 × 1024 frame and 600 Hz scanning speed. Microscope scanner settings were maintained constant for image capture for all samples. Images were analysed with ImageJ (NIH, http://rsb.info.nih.gov/ij) and the intensity of cellular immunofluorescence was quantified with the LAS Lite program (Leica Microsystems, Wetzlar, Germany).

### Immunoprecipitation assays

Cells were lysed as previously reported^19^,^38^,^65^. Immunoprecipitations were performed with *Gammabind G-Plus Sepharose* beads (Amersham Biosciences) as described^22^,^27^,^64^. Briefly for immunoprecipitation experiments, cells were grown in 100-mm dishes and were transfected with different plasmids tagged with myc-Flag or HA epitopes. The amount and type of the specific plasmid is indicated in each individual experiment. Whole cells extracts prepared 36 hours after transfection were lysed in lysis buffer containing 50 mM Tris-HCl (pH 7.5), 150 mM NaCl, 1% Nonidet, 1 mM EDTA, 1 mM sodium orthovanadate, 1 mM NaF and protease inhibitors as follows: 10 μg/mL leupeptin, 10 μg/mL aprotinin, 1 mM PMSF. Extracts were incubated on ice for 20 min and precleared by centrifugation at 14,000 rpm for 20 min at 4 °C. Then, extracts were immunoprecipitated with the specific antibody for each experiment. Immune complexes were recovered with γ-Bind G Sepharose (GE Healthcare Pharmacia). Beads were washed five times with lysis buffer and subjected to electrophoresis followed by immunoblot analysis with the corresponding antibodies indicated in each experiment.

### Immunohistochemistry

Biopsies from normal human amygdala were obtained with informed consent, fixed in formalin and embedded in paraffin. Immunodetection was performed with the DAKO EnVision Visualization Method (DAKO) with diaminobenzidine chromogen. Sections were counterstained with haematoxylin. VRK1 was detected with the VC polyclonal antibody (1:30) and Sox2 with monoclonal antibody Sox2 (E-4, 1:30). For immunofluorescence, the tissue sections, after antigen retrieval, and incubated with both the primary antibodies together (1:30 each one). The sections were incubated with the secondary antibodies α-mouse Cy3 (1:100) (Jackson ImmunoResearch) and α-rabbit Alexa Fluor 488 (1:100) (Molecular Probes; Eugene, OR). Cells were mounted with Vectashield (Vector Laboratories, Burlingame, CA) and analyzed with a LEICA SP5 DMI-6000B (Leica Microsystems) as reported^28^,^38^.

### Kinase assay

An *in vitro* kinase assay using GST-VRK1 or kinase-dead GST-VRK1(K179E) purified from bacteria was used in the assay with immunoprecipitated myc-Sox2 as substrate. The conditions for the *in vitro* kinase assay as reported^66^. Briefly VRK kinase activity was determined by assaying protein phosphorylation in a final volume of 30 μL containing kinase buffer (20 mM Tris-HCl pH 7.5, 5 mM MgCl_2_, 0.5 mM DTT and 150 mM KCl), 5 μM ATP and 5 μCi of [γ^32^P]ATP with 2 μg of GST-VRK1 and the immunoprecipitated Sox2 protein. The proteins in the assay were analysed by electrophoresis in 12.5% SDS-polyacrilamide gels. The gels were stained with Coomassie Blue or proteins were transferred to PVDF membrane and the incorporated activity was measured^66^.

### Immunoblots

Protein extracts were quantified using the Protein assay kit (Bio-Rad; Hercules, CA). Protein were fractionated in a SDS polyacrylamide gel and transferred to a PVDF Immobilon-P or Immobilon-FL membranes (Millipore) as reported^24^,^38^,^58^. PDVF membranes were blocked for 1 hour, or overnight, at room temperature with 5% defatted milk in TBS-T buffer (25 mM Tris-HCl pH 8, 50 mM NaCl, 2,5 mM KCl, 0,1% Tween-20). Membranes were incubated with the primary antibody for 1 hour at room temperature or overnight at 4 °C followed by extensive washing in TBS-T buffer. Next, membranes were incubated with the corresponding secondary antibody for 30 minutes at room temperature followed by washing. Membrane signals were detected and quantified using in a Li-Cor Odyssey system (Thermo-Fisher)^28^. Alternatively, some western blotss were also analysed with the ECL Western Blotting Detection Reagents” (GE Healthcare) and luminescence detected using X-ray film.

### qRT-PCR

RNA (100 ng) was used in a one-step qRT-PCR using the t *iScript*^*TM*^ *One-Step RT-PCR Kit With SYBR*^*®*^ *Green* (Bio-Rad). The primers used were: Sox2 (forward: 5′-CCCGGATCCGCCATGTACAACATGATGGAGACGGAGCTG-3′; reverse: 5′-CCCGAATTCGCCTCACATGTGTGAGAGGGGCAGTGTGCC-3); VRK1 (forward: 5′-CCAACGAGCTGCAAAACC-3′; reverse: 5′-TGTCATGTAGACCAGACCCCC-3′); Oct4 (forward: 5′-AAGCGATCAAGCAGCGACTAT-3′; reverse: 5′- GGAAAGGGACCGAGGAGTACA-3′); GAPDH (forward: 5′-GGTCTTACTCCTTGGAGGCCATGT-3′; reverse: 5′-ACCTAACTACATGGTTTACATGTT-3′); macroH2A2 Primers: macroH2A2 (forward: 5′-TGCTGGCAGTTGCCAATGACGA-3′; reverse: 5′-CGTTTCCGACTTGCCTTTGGTC-3′; macroH2A1.2 Primers (forward: 5′- CTGAACCTTATTCACAGTGAAATCAGT-3′; reverse: 5′- GTGTTTCCTAGGTCATCTTTAAGGT - 3′).

### Luciferase assay

MDA-MB-231 and HEK293T cells were transfected with different amounts of Sox2, tagged with myc-Flag (pCMV6-myc-Flag-Sox2) and pGL2-B-VRK1 (−1028 + 52) or 0.5 μg of pGL2-B-VRK1 (−264 + 52) vectors. Cyclin D1 promoter: pA3- Cyclin D1(−1745 + 134)-Luc^59^. pGL2-basic was used as negative control. Luciferase activity was determined with a Dual luciferase reporter system from Promega (Madison, WI) following the manufacturer instructions as previously reported^29^,^67^.

### Retinoic acid differentiation

For all-trans retinoic acid (RA) (Sigma-Aldrich) differentiation of NT2, 1.6 × 10^5^ cells were seeded in 100 mm culture dishes and treated during 14 days with 10 μM RA as previous described^61^.

### Database of protein interactions

The protein interactions from this publication have been submitted to the IMEx (http://www.imexconsortium.org) consortium through IntAct database^68^ and assigned the identifier IM-24906.

## Additional Information

**How to cite this article**: Moura, D. S. *et al*. Oncogenic Sox2 regulates and cooperates with VRK1 in cell cycle progression and differentiation. *Sci. Rep.* **6**, 28532; doi: 10.1038/srep28532 (2016).

## Supplementary Material

Supplementary Information
