# Supplementary material for: Oncogenic Sox2 regulates and cooperates with VRK1 in cell cycle progression and differentiation
Source: Sci Rep. 2016 Jun 23;6:28532. doi: 10.1038/srep28532 (PMC4917848; doi:10.1038/srep28532)
Supplement: Supplementary Information [file srep28532-s1.pdf]

Supplementary Information

Oncogenic Sox2 regulates and cooperates with VRK1 in cell cycle progression and differentiation

David S. Moura [1,2], Isabel F. Fernández [1], Gema Marín-Royo [1], Inmaculada López-Sánchez [1], Elena Martín-Doncel [1,2], Francisco M. Vega [3,4] and Pedro A. Lazo [1,2]

[1] *Experimental Therapeutics and Translational Oncology Program, Instituto de Biología Molecular y Celular del Cáncer, Consejo Superior de Investigaciones Científicas (CSIC)-Universidad de Salamanca, Campus Miguel de Unamuno, 37007 Salamanca, Spain.*

[2] *Instituto de Investigación Biomédica de Salamanca-IBSAL, Hospital Universitario de Salamanca, 37007 Salamanca, Spain*

[3] *Instituto de Biomedicina de Sevilla (IBIS), CSIC-Universidad de Sevilla-Hospital Universitario Virgen del Rocío, 41013 Sevilla, Spain*

[4] *Departamento de Fisiología Médica y Biofísica, Facultad de Medicina, Universidad de Sevilla, 41013 Sevilla, Spain*

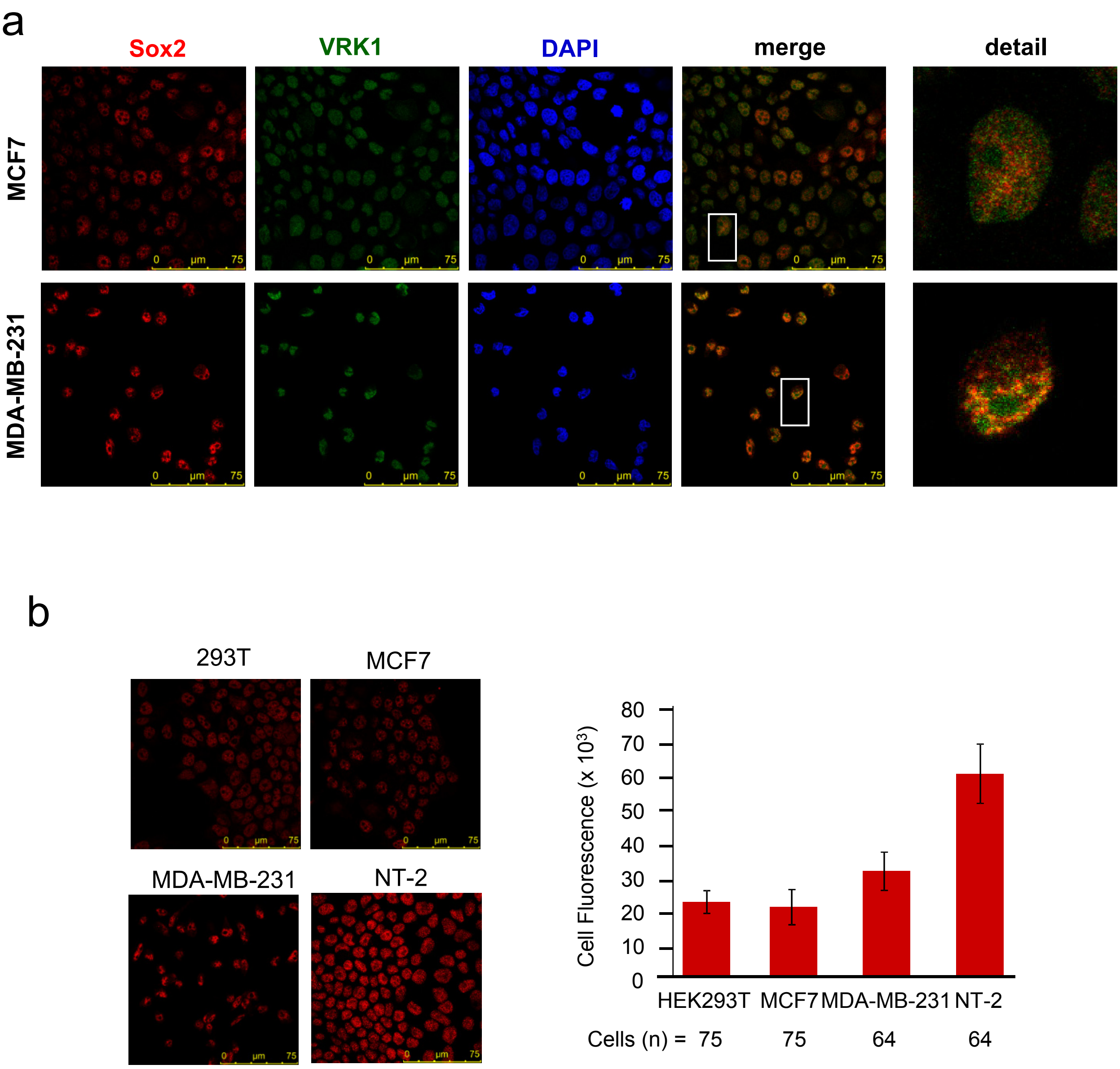

Supplementary Figure S1. a. Nuclear colocalization of Sox 2 and VRK1 in nuclei of MCF7 and MDA-MB-231 cells was detected by confocal immunofluorescence microscopy. Sox2 (murine monoclonal antibody) and VRK1 (rabbit polyclonal VC1). **b**. Level of nuclear Sox2 protein in four cell lines cell lines. Nuclear fluorescence was quantified with the image J program. In the graph to the right is shown the relative level in each cell line. The mean and standard deviations are shown.

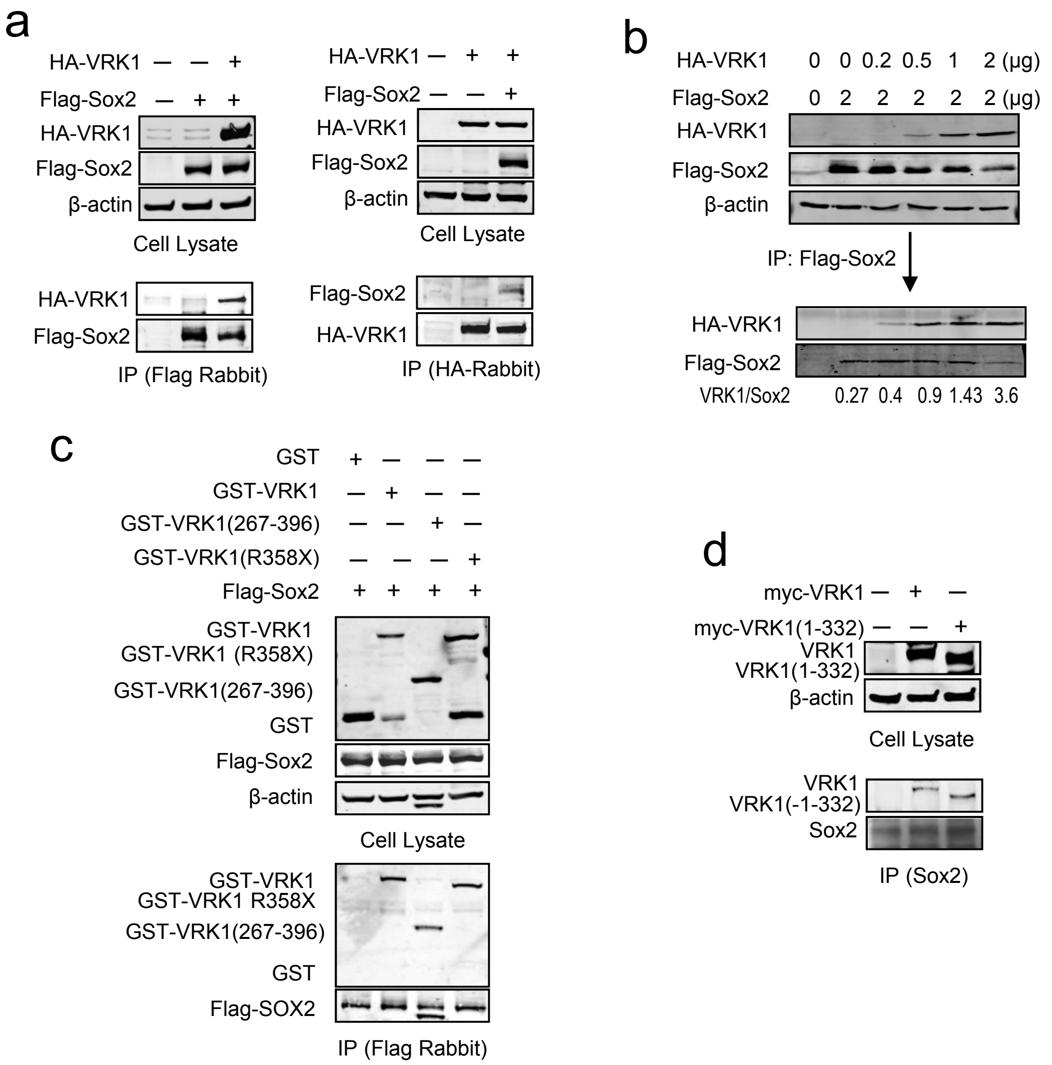

Supplementary Figure S2. A. Sox2 interacts with VRK1. a. The transfected VRK1 and SOX2 interact one with each other, being the interaction dependent of Sox2 levels. The 293T cells were transfected, with Sox2 (pCMV6-Flag-myc-Sox2), with wild-type VRK1 (pCEFL HA-VRK1). 48 later the cells were lysed and the extracts immunoprecipitated with a rabbit polyclonal anti-Flag (F7425) antibody or with rabbit polyclonal anti-HA (H6908) antibody. The immunoprecipitates were loaded in a 10% acrylamide gel. HA-VRK1 was detected with anti-HA mAb (16B12). Endogenous VRK1 was detected using a anti-VRK1 (1B5) antibody. Flag-tagged Sox2 was detected with anti-Flag murine mAb (F4042) antibody (1:1000). Human β-Actin was detected using a mouse monoclonal anti-β-Actin antibody. **b**. Dose dependent interaction between Sox2 and VRK1. The 293T cells were transfected, with Sox2 (pCMV6-Flag-myc-Sox2), with increasing amounts of wild-type VRK1 (pCEFL HA-VRK1). Sox2 was immunoprecipitated and the presence of VRK1 determined as in part a. **c**. The 293T cells were transfected with Sox2 (pCMV6-Flag-myc-Sox2), and different constructions of VRK1 protein: GST-tagged VRK1 (pGEX-GST-VRK1), carboxy terminal GST-VRK1(267-369) and truncated VRK1 (pGEX-GST-VRK1 (R358X). 48 hours later the cells were lysed and the extracts were immunoprecipitated with a polyclonal anti-flag (F7425) antibody. The immunoprecipitates were loaded in a 10% acrylamide gel. Wild type GST-VRK1, GST-VRK1 carboxy-terminal and GST-VRK1(R358X) were detected using a mouse monoclonal anti-GST (B-14). Human SOX2 was detected using a mouse monoclonal anti-flag (F4042 β-Actin was detected using a mouse monoclonal anti-β-Actin antibody. **d**. The 293T cells were transfected with myc-tagged, wild-type VRK1 (pcDNA3.1-VRK1) and amino terminal VRK1 (pcDNA3.1-VRK1(1-332)). 48 hours later the cells were lysed and the extracts were incubated with a mouse monoclonal anti-Sox2 (E-4) antibody overnight. After the immunoprecipitation, the extracts were loaded in a 10% acrylamide gel. Wild-type VRK1 and VRK1 amino terminal were detected using a rabbit polyclonal anti-myc, endogenous Sox2 was detected using a goat polyclonal (Y-17) antibody and human β-Actin was detected using a mouse monoclonal anti-β-actin antibody.

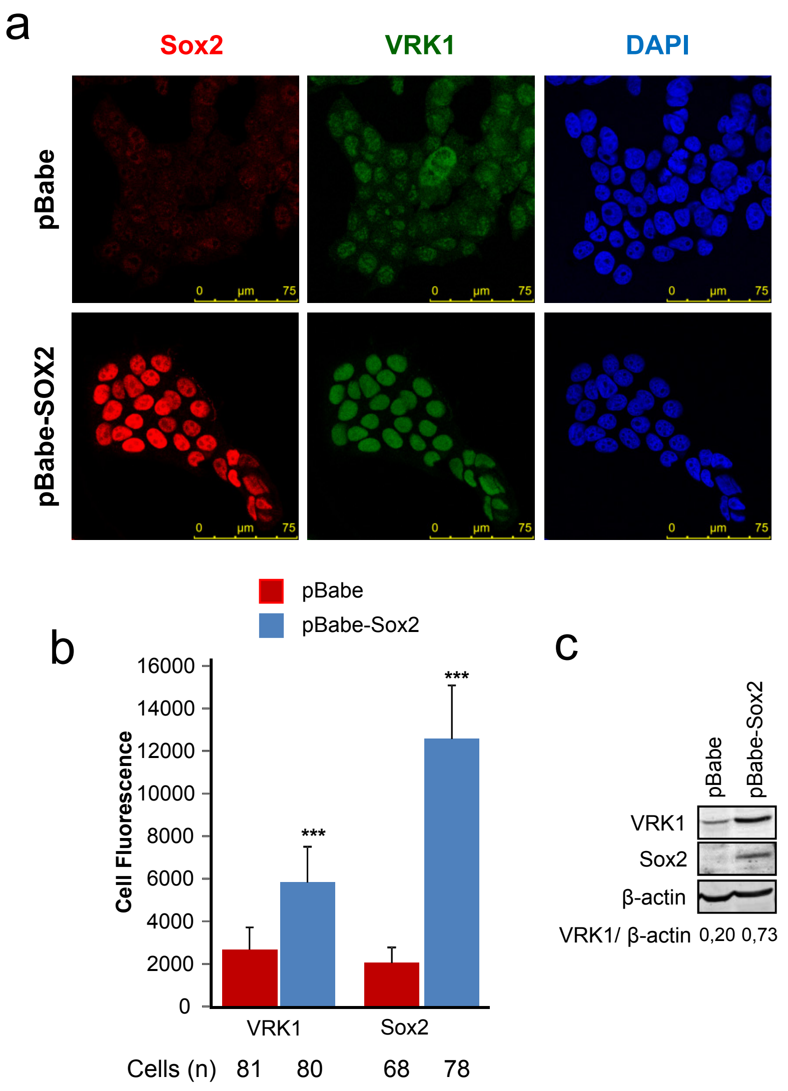

Supplementary Figure S3. **Sox2 activates endogenous *VRK1* gene expression in MCF7 cells. a.** Effect of overexpression of Sox2 expressed in breast cancer MCF7 cells that were infected with the retroviral plasmid pBabe-Sox2. Cells were studied by confocal immunofluorescence. The quantification of the fluorescence is shown in the graph to the right. **b.** Fluorescence of nuclear VRK1 protein after infection with empty vector (pBabe) or with pBabe-Sox2. **c.** Effect of Sox2 overexpression in MCF7 cells on endogenous VRK1 protein determined in cell lysates by immunoblot.

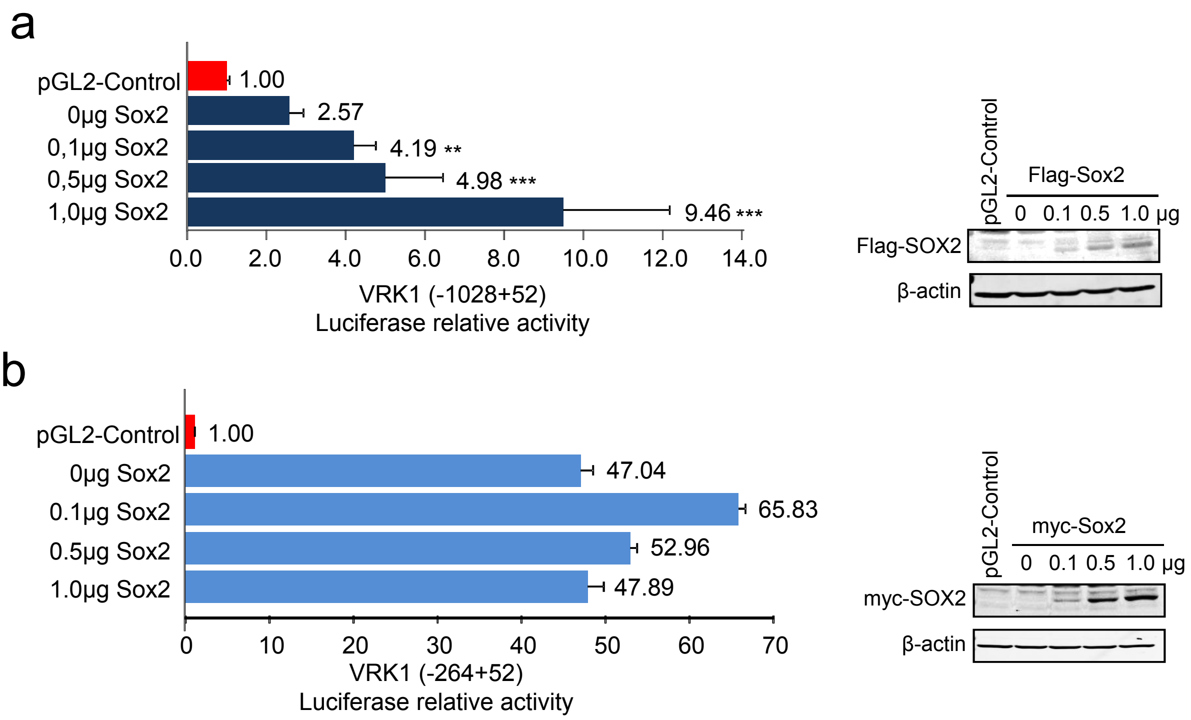

Supplementary Figure S4. Sox2 activates the human *VRK1* gene promoter. a. Dose-dependent activation of the VRK1 gene promoter by expression of Sox2. Two plasmids expressing regions from the long proximal VRK1 promoter (-1028 to + 52) cloned in pGL2-b-luciferase vector and Renilla-luciferase as internal control were transfected in HEK293T cells with different amounts of plasmid Flag-2-Sox2. **b.** Lack of Dose-dependent activation of the VRK1 gene short promoter (-264 to + 52), cloned in pGL2-b-luciferase vector, by expression of increasing amounts of Sox2. Renilla-luciferase was used as internal control. The PGL2-Control is a plasmid expressing luciferase under the SV40 promoter used as positive control. pGL2-basic,, a promoterless plasmid, was used as negative and background control. Plasmid expression was also confirmed by immunoblot. Human β-actin was detected using a mouse monoclonal anti-β-actin antibody (Sigma-Aldrich; St. Louis, MO, USA) (1:5000). (Student's Test:*<0,05; **<0,005; ***<0,0005).

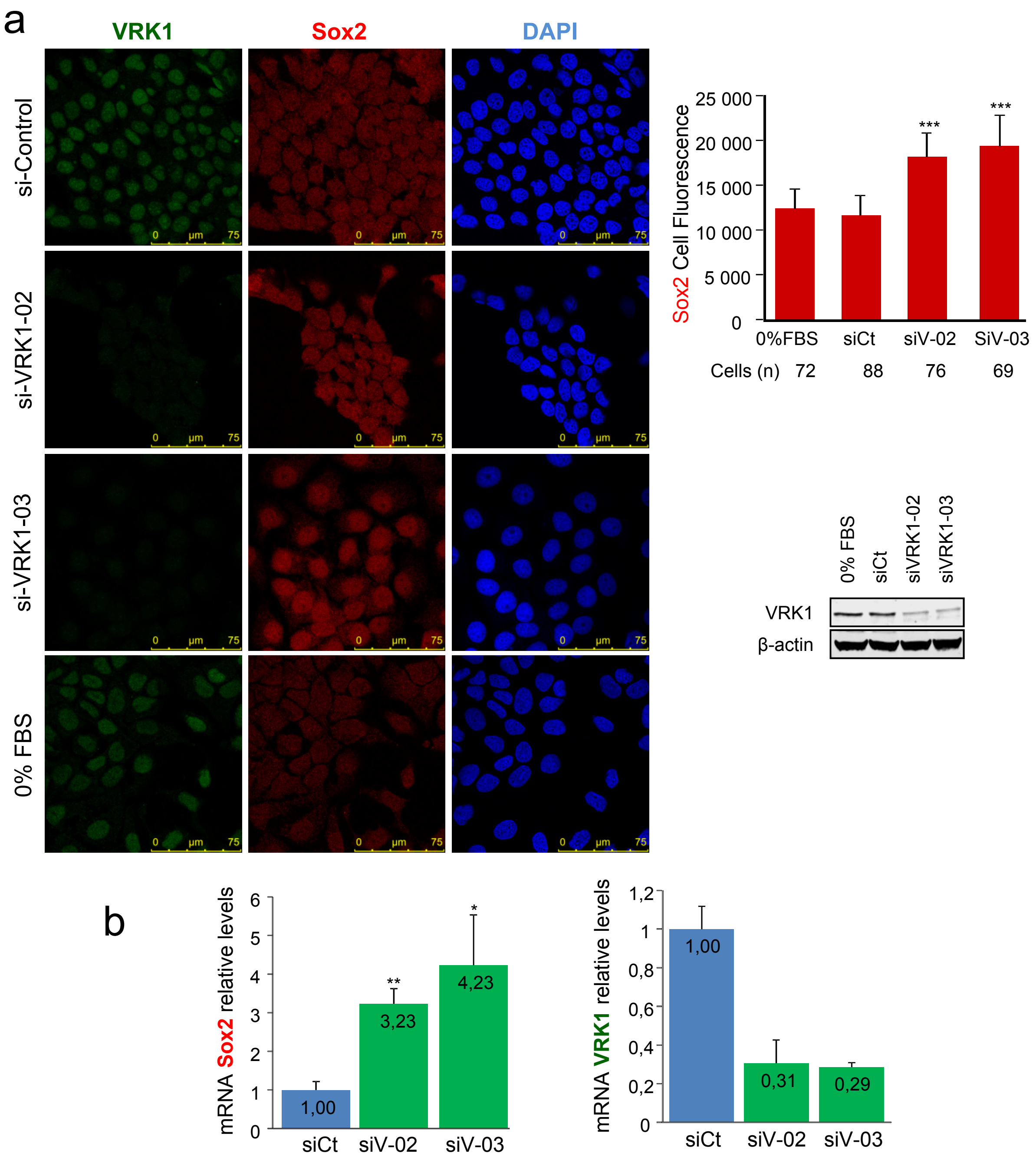

Supplementary Figure S5. **Effect of VRK1 knockdown on Sox2 in MCF-7 cells. a.** Sox2 protein and fluorescence levels increase when VRK1 levels decrease in MCF7 cells. In immunofluorescence, human endogenous Sox2 was detected with a mouse monoclonal anti-Sox2 (E-4). Human endogenous VRK1 was detected using a rabbit polyclonal anti-VRK1 antibody. Human endogenous VRK1 was detected, by Western Blot, using a murine mAb anti-VRK1 (1B5). Human endogenous Sox2 was detected using a goat polyclonal anti-Sox2 (Y-17). and human β-actin was detected using a mouse monoclonal anti-β-Actin antibody. **b**. Depletion of VRK1 results in an increase of Sox2 mRNA. 72 hours after siControl, si-VRK1-02 and si-VRK1-09 treatment, was performed the fixation and permeabilization of MCF-7 cells. For expression analysis, MCF-7 cells were transfected with siControl (siCt), siVRK1-02 (siV-02) or siVRK1-03 (siV-03) and 72hours later the mRNA was extracted. The qRT-PCR was performed using primers for VRK1, Sox2 and GAPDH.

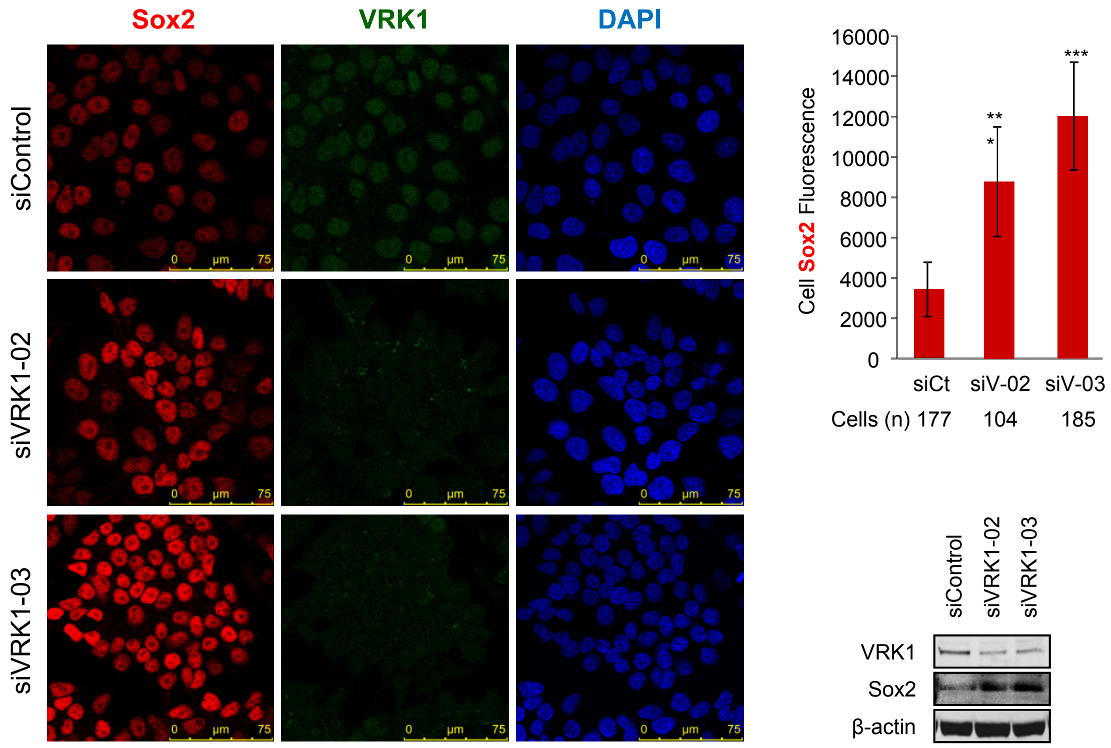

Supplementary Figure S6 – Effect of VRK1 knockdown on the Sox2 levels in NT2 cells. 72 hours after siControl (siCt), si-VRK1-02 (siV-02) and si-VRK1-03 (siV-03) treatment, was performed the fixation and permeabilization of Ntera-2 cells. By immunofluorescence, human endogenous Sox2 was detected with a rabbit polyclonal anti-Sox2 antibody. Human endogenous VRK1 was detected using a rabbit polyclonal anti-VRK1 antibody. DAPI (1:1000). For immunoblot analysis, cells were harvested, lysed and loaded in a 10% acrylamide gel. Human endogenous VRK1 was detected using an anti-VRK1murine mAb (1B5) antibody. Human endogenous Sox2 was detected using a rabbit polyclonal anti-Sox2 antibody, and human β-actin was detected using a murine mAb anti-β-actin antibody.

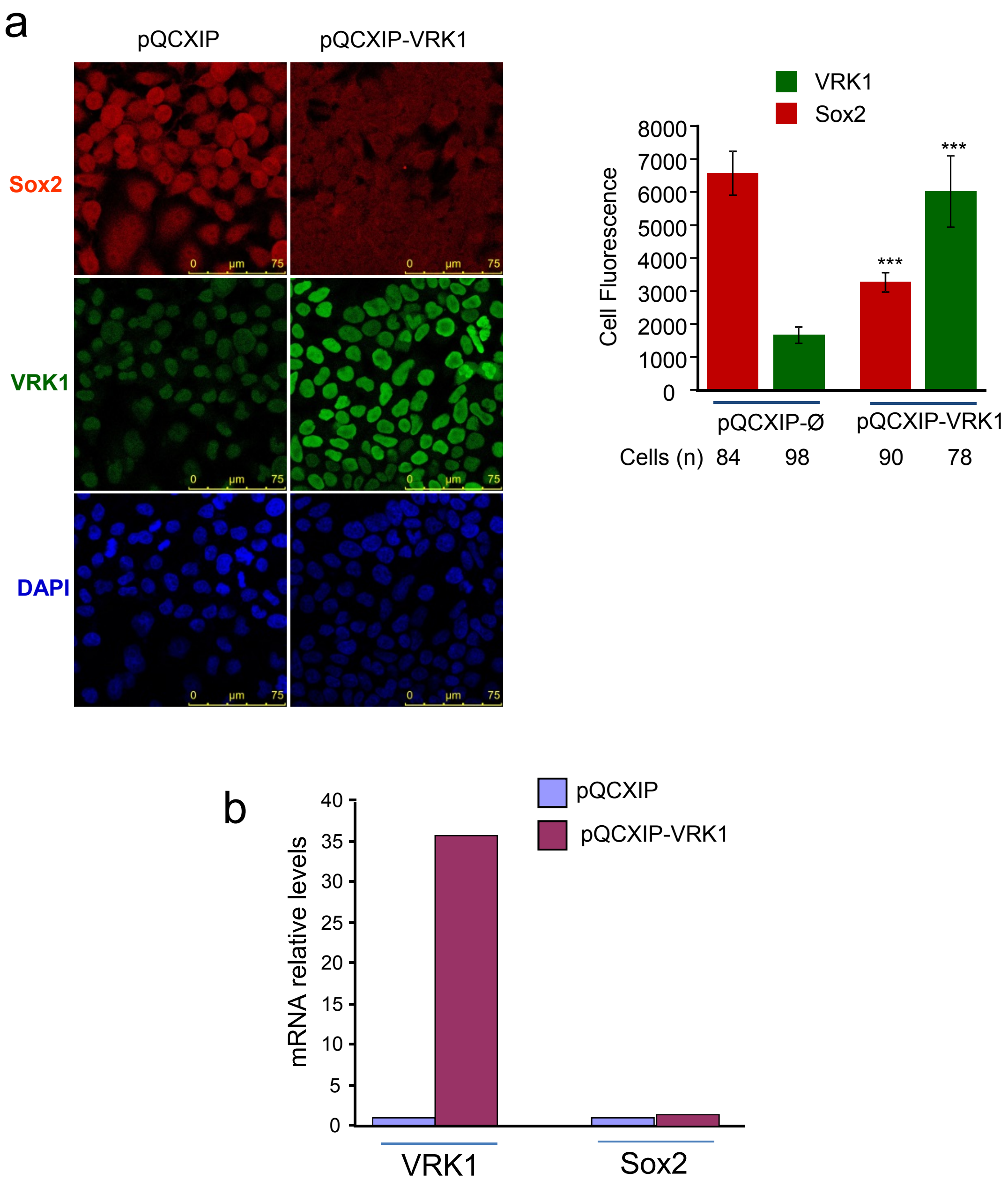

Supplementary Figure S7. Effect of VRK1 overexpression on Sox2 gene expression in HeLa cells. HeLa cells were infected with a retroviral plasmid (pQCXIP) or a construct expressing VRK1 (pQCXIP-VRK1). Cells were grown in the presence of puromycin and used as a pool.. **a**. Detection of the nuclear levels of VRK1 and Sox2 detected by confocal immunofluorescence. To the right is shown the quantification of individual cells. **b**. Effect of VRK1overexpression on Sox2 mRNA in a stable HeLa cells used as a pool. Total RNA was extracted from stable cell lines infected with either pQCXIP (control vector) or pQCXIP-VRK1. This RNA was used for quantification by qRT-PCR with VRK1 and Sox2 specific primers. Result is the mean of two experiments.

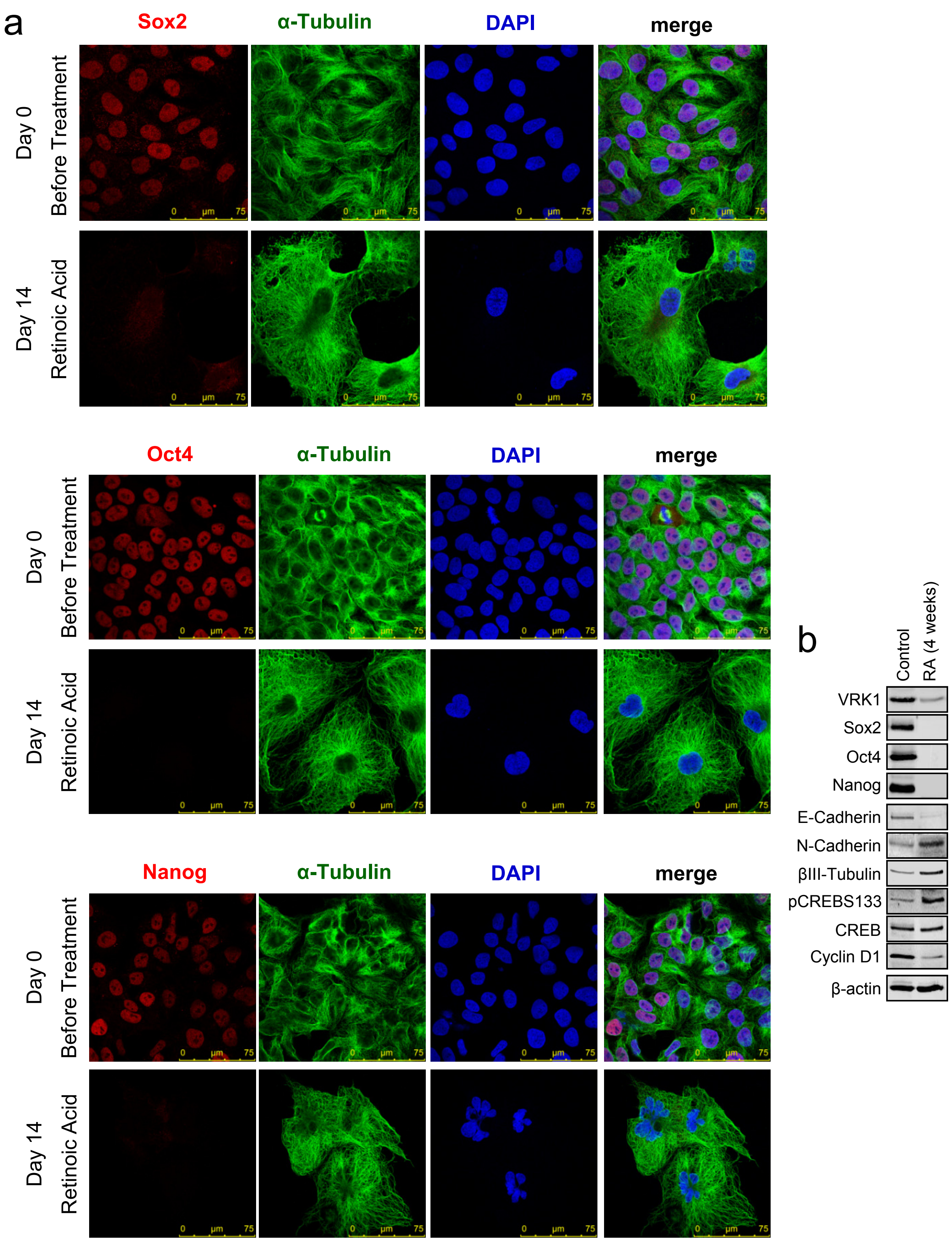

Supplementary Figure S8. **Diferentiation of NT2 cells induced by retinoic acid**. **a**.Loss of expression of Sox2, Oct4 and nanog in RA differentiated NT2 cells by confocal microscopy (left column). Reorganization of α-tubulin induced by retinoic acic (second column). **b**. Detection of several markers in non-treated and treated(differentiated cells).

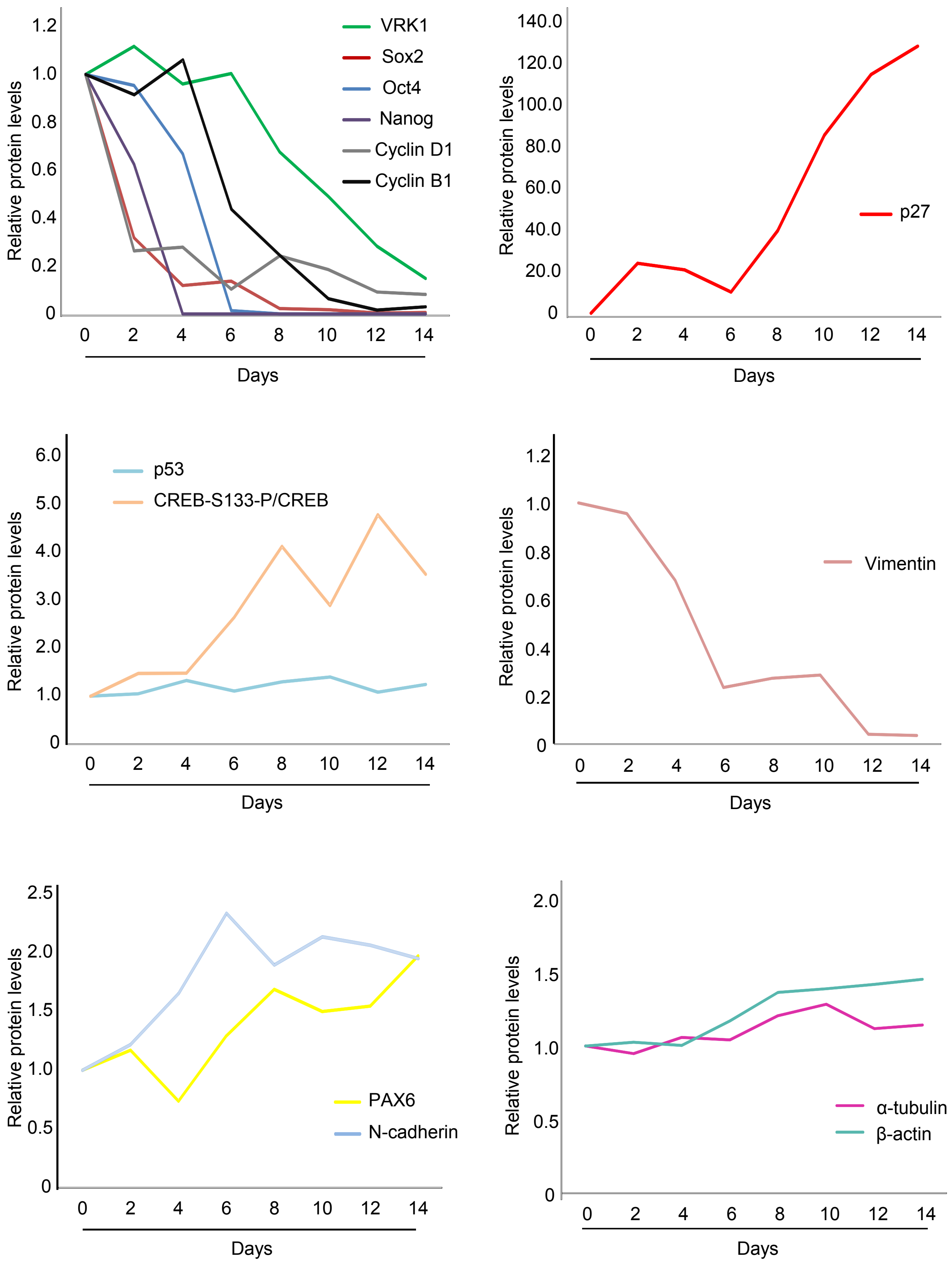

Supplementary Figure S9. Quantification of the immunoblots in Figure 7 showing the differentiation of NT-2 cells induced by RA.
